# Fumonisins affect the intestinal microbial homeostasis in broiler chickens, predisposing to necrotic enteritis

**DOI:** 10.1186/s13567-015-0234-8

**Published:** 2015-09-23

**Authors:** Gunther Antonissen, Siska Croubels, Frank Pasmans, Richard Ducatelle, Venessa Eeckhaut, Mathias Devreese, Marc Verlinden, Freddy Haesebrouck, Mia Eeckhout, Sarah De Saeger, Birgit Antlinger, Barbara Novak, An Martel, Filip Van Immerseel

**Affiliations:** Department of Pathology, Bacteriology and Avian Diseases, Faculty of Veterinary Medicine, Ghent University, Salisburylaan 133, 9820 Merelbeke, Belgium; Department of Pharmacology, Toxicology and Biochemistry, Faculty of Veterinary Medicine, Ghent University, Salisburylaan 133, 9820 Merelbeke, Belgium; Department of Applied Biosciences, Faculty of Bioscience Engineering, Ghent University, Valentin Vaerwyckweg 1, 9000 Ghent, Belgium; Department of Bio-analysis, Faculty of Pharmaceutical Sciences, Ghent University, Ottergemsesteenweg 460, 9000 Ghent, Belgium; Biomin Research Center, Technopark 1, 3430 Tulln, Austria

## Abstract

Fumonisins (FBs) are mycotoxins produced by *Fusarium* fungi. This study aimed to investigate the effect of these feed contaminants on the intestinal morphology and microbiota composition, and to evaluate whether FBs predispose broilers to necrotic enteritis. One-day-old broiler chicks were divided into a group fed a control diet, and a group fed a FBs contaminated diet (18.6 mg FB1+FB2/kg feed). A significant increase in the plasma sphinganine/sphingosine ratio in the FBs-treated group (0.21 ± 0.016) compared to the control (0.14 ± 0.014) indicated disturbance of the sphingolipid biosynthesis. Furthermore, villus height and crypt depth of the ileum was significantly reduced by FBs. Denaturing gradient gel electrophoresis showed a shift in the microbiota composition in the ileum in the FBs group compared to the control. A reduced presence of low-GC containing operational taxonomic units in ileal digesta of birds exposed to FBs was demonstrated, and identified as a reduced abundance of *Candidatus* Savagella and *Lactobaccilus* spp. Quantification of total *Clostridium perfringens* in these ileal samples, previous to experimental infection, using *cpa* gene (alpha toxin) quantification by qPCR showed an increase in *C. perfringens* in chickens fed a FBs contaminated diet compared to control (7.5 ± 0.30 versus 6.3 ± 0.24 log10 copies/g intestinal content). After *C. perfringens* challenge, a higher percentage of birds developed subclinical necrotic enteritis in the group fed a FBs contaminated diet as compared to the control (44.9 ± 2.22% versus 29.8 ± 5.46%).

**Electronic supplementary material:**

The online version of this article (doi:10.1186/s13567-015-0234-8) contains supplementary material, which is available to authorized users.

## Introduction

Mycotoxins are naturally occurring secondary fungal metabolites produced both pre- and post-harvest in crops and other feed and food commodities. *Fusarium*, *Aspergillus*, and *Penicillium* are the most abundant mycotoxin producing mould genera contaminating feed and feed raw materials [1]. Fumonisins (FBs) are produced by *Fusarium verticillioides*, *F. proliferatum*, and other *Fusarium* species and are among the most widespread mycotoxins [2]. FBs are ubiquitous contaminants of corn and other grain products [3]. A global survey on the occurrence and contamination levels of mycotoxins in feed raw materials and finished feed for livestock animals showed that 54% of 11 439 tested samples were contaminated with FBs [4]. FBs were most frequently detected in South American (77%), African (72%) and Southern European (70%) samples, and less frequently in Oceania (10%) [4]. The economic impact of in animal feed is rather difficult to measure because information about subclinical effects on animal health and productivity losses due to chronic low level exposure is limited. Wu [5] estimated the annual economic losses in the USA due to FBs in animal feed to be US$ 1–20 million and US$ 30–46 million, in a normal and an outbreak year of *Fusarium* ear rot, respectively.

More than 28 FB homologues have been identified. Fumonisin B_1_ (FB_1_) is the most common and the most thoroughly investigated mycotoxin because of its toxicological importance. FB_2_ and FB_3_ are less prevalent, and differ structurally from FB_1_ in the number and position of hydroxyl groups [2]. FBs competitively inhibit the ceramide synthase and, as a result, interfere with the biosynthesis of ceramides and sphingolipids of cell membranes [2,6]. Clinical outbreaks have been reported in horses (equine leucoencephalomalacie, ELEM) and pigs (porcine pulmonary edema, PPE). These animal species are regarded as the most susceptible to the effects of FBs [2]. In general, poultry are considered to be quite resistant toward the deleterious effects of FBs. Also species differences occur, laying hens and broilers are less sensitive to FBs compared to turkeys and ducks [7-11]. In broilers, systemic uptake of FB_1_ after oral exposure is low, indicating that the absorption is negligible [12]. Following the consumption of FBs contaminated feed, the intestine is the first organ to be exposed to these toxins and negative effects on intestinal tissues have been reported [13]. The jejunum of broilers exposed to high FB_1_ concentrations (≥100 mg/kg feed) for 28 days displays a reduced villus height (VH) and villus-to-crypt ratio (V:C) [14]. Besides a mild villus atrophy, also goblet cell hyperplasia is observed in broiler chicks exposed to high levels of FB_1_ (300 mg/kg feed) for 2 weeks [15]. It has been shown in vitro that FB_1_ has a toxic effect on both undifferentiated and differentiated porcine intestinal epithelial cells (IPEC-1). The effect of FB_1_ on epithelial cell proliferation correlates with a cell cycle arrest in the G0/G1 phase [16]. A negative effect of FB_1_ on the expression of cell junction proteins E-cadherin and occludin, and consequently on the intestinal epithelial integrity, has been shown in vivo in pigs [17,18]. Furthermore, FB_1_ modulates intestinal immunity by decreasing the expression of several cytokines, for example interleukin (IL)-1β, IL-2, IL-8, IL-12p40 and interferon (IFN)-γ in pigs [19-21]. It has been shown that exposure of pigs to 0.5 mg FB_1_/kg bodyweight (BW) for 6 days enhanced intestinal colonization and translocation of a septicemic *Escherichia coli* (SEPEC) [20]. Feeding a FBs contaminated diet (11.8 mg FB_1_+FB_2_/kg feed) for 9 weeks transiently modified the faecal microbiota composition in pigs. Co-exposure to FBs and *Salmonella* Typhimurium amplified this phenomenon [22]. At present it is unclear what may be the consequences of long term exposure to low levels of FBs.

In poultry, necrotic enteritis (NE) is caused by netB producing *Clostridium perfringens* strains. *C. perfringens* is a Gram-positive spore-forming anaerobic bacterium that is commonly found in the environment and the gastro-intestinal tract of animals and humans as a member of the normal microbiota [23-25]. NE in chickens is still an important intestinal disease despite the application of preventive and control methods, including coccidiosis control. The acute form of the disease causes mortality without premonitory symptoms. The more frequently occurring subclinical form is characterized by intestinal mucosal damage without clinical signs or mortality, leading to decreased performance [24,26]. However, healthy birds often carry netB positive *C. perfringens* without showing any clinical symptoms of NE [24]. An outbreak of NE is a complex process requiring one or a number of predisposing factors rather than just the presence of pathogenic *C. perfringens* [27-29]. Pre-existing mucosal damage caused by coccidiosis, high protein feed (including fishmeal) and indigestible non-starch polysaccharides are well known predisposing factors [29]. Recently, it was shown that the mycotoxin deoxynivalenol (DON) is also a predisposing factor for the development of NE, through damage to the epithelial barrier and an increased intestinal nutrient availability for clostridial proliferation [30]. Although FBs are ubiquitous contaminants in poultry feed, information about their impact on the intestinal microbial homeostasis in broiler chickens is lacking.

The objective of this study was to evaluate the effect of FBs on the intestinal microbial homeostasis, at concentrations approaching the European Union maximum guidance levels (20 mg FB_1_+FB_2_/kg feed) [31]. Therefore, the influence of FBs on the intestinal microbiota composition and intestinal morphology was investigated. In addition, an attempt was made to demonstrate the consequences of the effect of FBs on the intestinal microbial homeostasis in a subclinical necrotic enteritis model.

## Material and methods

### Fumonisins

FBs (8.64mg FB_1_+FB_2_/g culture material) (Biopure - Romer Labs Diagnostic GmbH, Tulln, Austria) were produced in vitro from a culture of *F. verticillioides,* and subsequently crystallized [32]. For the in vitro assessment of the impact of FB_1_ on growth and toxin production characteristics of *C. perfringens*, serial dilutions were prepared in tryptone glucose yeast (TGY) broth medium of a FB_1_ stock solution of 5000 μg/mL (Fermentek, Jerusalem, Israel) that had been prepared in anhydrous methanol and stored at −20 °C.

### Bacterial strain and growth conditions

*C. perfringens* strain 56, a *netB*^*+*^ type A strain, was originally isolated from a broiler chicken with NE and has been shown to be virulent in an in vivo infection model [30,33]. The inoculum for the oral infection of chickens and in vitro experiments was prepared by culturing *C. perfringens* anaerobically overnight at 37 °C in brain heart infusion broth (BHI, Bio-Rad, Marnes-la-Coquette, France) or tryptone glucose yeast (TGY) broth medium, respectively. The colony forming units of *C. perfringens*/mL was assessed by plating tenfold dilutions on Colombia agar (Oxoid, Basingstoke, UK) with 5% sheep blood, followed by anaerobic overnight incubation at 37 °C.

### Animal experiment

#### Birds and housing

The animal experiment was performed using non-vaccinated Ross 308 broiler chickens, obtained as one-day-old chicks from a commercial hatchery (Vervaeke-Belavi, Tielt, Belgium). Animals of both treatment groups, control diet and FBs contaminated diet, were housed in the same temperature controlled room, in pens of 1.44 m^2^, on wood shavings. Each group consisted of three pens of 34 birds, with approximately equal numbers of males and females. Animal units were separated by solid walls to prevent direct contact between animals from different pens. All cages were decontaminated with peracetic acid and hydrogen peroxide (Metatectyl HQ, Metatecta, Kontich, Belgium) and a commercial anticoccidial disinfectant (Bi-OO-Cyst Coccidial Disinfectant, Biolink, York, UK) prior to the housing of the chickens. Water and feed was provided *ad libitum*. Chickens were not fasted before euthanasia. The animal experiment was approved by the Ethical Committee of the Faculty of Veterinary Medicine and Bioscience Engineering, Ghent University (EC 2012/194).

#### Feed preparation and experimental diets

All chickens were fed a starter diet during the first eight days of the trial, and subsequently a grower diet. The diet was wheat and rye based, with soybean meal as main protein source during the first 16 days. From day 17 onwards, the same grower diet was fed with the exception that fishmeal replaced soybean meal as main protein source [30,33].

FBs contaminated feed was produced by adding lyophilized FBs culture material to a control diet. Mycotoxin contamination of both the control and FBs contaminated diet was analyzed by a validated multi-mycotoxin liquid chromatography-tandem mass spectrometry method (LC-MS/MS) [34]. Three different batches of FBs contaminated feed were produced: a starter diet, a grower diet with soybean meal and a grower diet with fishmeal, respectively. Therefore, FBs culture material was added to 500 g of the corresponding batches of control diet. For each batch, this premix was then mixed with 5 kg of control feed to assure homogeneous distribution of the toxin and finally mixed for 20 min in the total amount of feed needed for each batch. To test mycotoxin contamination, samples were taken at three different locations in each batch, subsequently pooled per batch and analyzed for mycotoxin contamination as described above. Trace amounts of nivalenol and DON were detected in the control feed (0.059-0.116 and 0.113-0.170 mg/kg feed, respectively). Analyzed mycotoxins, their limit of detection and limit of quantification were as previously described [30,34]. The levels of FBs and all other tested mycotoxins in the different batches of control feed were below the limit of detection. The average levels of FB_1_, FB_2_ and FB_3_ in the different batches of FBs contaminated feed were 10.4 mg/kg, 8.2 mg/kg and 2.0 mg/kg, respectively (Table 1). The average sum of FB1+FB2, 18.6 mg/kg feed was approaching the EU maximum guidance level in feed for poultry of 20 mg FB1+FB2/kg (2006/576/EC) [31].

**Table 1:** **Concentration of FB**_**1**_ **, FB**_**2**_ **and FB**_**3**_ **in different batches of FBs contaminated diet**

Type of feed	Feeding period	FB_1_	FB_2_	FB_1_+FB_2_	FB_**3**_
		mg/kg feed			
Starter	day 1-8	8.0	6.6	14.6	1.6
Grower (soybean meal)	day 9-16	14.5	10.6	25.1	2.5
Grower (fishmeal)	day 17-end	8.8	7.4	16.2	1.8

### Evaluation of the impact of FBs on broiler health

The BW of all chickens was measured on day 1 and day 8. On day 15, six birds (3♂/3♀) per pen were euthanized using an overdose of sodium pentobarbital (Natrium Pentobarbital 20%, Kela Veterinaria, Sint- Niklaas, Belgium). A blood sample was collected and subsequently a necropsy was performed. Blood samples were centrifuged (2851 × *g*, 10 min, 4 °C) and plasma was stored at ≤−20 °C until sphinganine (Sa) and sphingosine (So) concentrations were analyzed. Sa/So is suggested to be the most sensitive biomarker to FBs intoxication in many animals [2]. Plasma Sa and So concentrations were analyzed by a commercial service provider (Biocrates Life Sciences AG, Innsbruck, Austria). Briefly, Sa and So were extracted from plasma and measured in the presence of internal standards using LC-MS/MS with electrospray ionization.

The BW and weight of different organs (liver, spleen, kidneys, proventriculus, ventriculus, bursa of Fabricius, heart and lungs) were recorded. The weight of each organ was converted to a relative percentage of the BW.

### Evaluation of the impact of FBs on intestinal morphology

After measuring the length of the different small intestinal segments, 1 cm samples from the mid-duodenum, mid-jejunum and mid-ileum were collected and fixed in neutral-buffered formalin. Small intestinal segments were defined as duodenum encompassing the duodenal loop, jejunum between the end of the duodenal loop and Meckels diverticulum and ileum between Meckels diverticulum and the ileo-cecal junction. Villus height and crypt depth of mid-duodenum, mid-jejunum and mid-ileum were measured on hematoxylin and eosin stained histological paraffin sections using light microscopy with Leica LAS software (Leica Microsystems, Diegem, Belgium). The average of ten measurements per segment per animal was calculated.

### Assessment of the impact of FBs on the intestinal microbiota

At day 15, intestinal content samples of the second half of the different small intestinal segments of all six bird per pen were collected, snap frozen in liquid nitrogen, and subsequently stored at −80 °C until further DNA extraction. DNA from intestinal content (duodenum, jejunum and ileum) was extracted using a modified QIAamp DNA Stool mini Kit (Qiagen, Hilden, Germany) protocol. An enzymatic pretreatment with lysozyme and a mechanical disruption step with a bead-beater was added to the original protocol. In brief, frozen intestinal content (250 mg) was transferred into a bead beating tube filled with 0.7 g of glass beads (Ø 100 μm), 0.6 g of ceramic beads (Ø 1.4 mm) and one glass bead (Ø 3.8 mm). Subsequently, 200 μL of TE buffer of pH 8 (10 mM Tris–HCl and 1 mM EDTA (Sigma Aldrich, Steinheim, Germany)) and 125 μL of freshly prepared lysozyme (100 mg/mL, Sigma Aldrich) was added. After homogenizing by vortex mixing (1 min), samples were incubated at 37 °C for 45 min at 1000 rpm on a Thermomixer compact shaker incubator (Eppendorf, Hamburg, Germany). The final volume was adjusted to 2 mL with ASL buffer (Qiagen) and samples were bead beated for 10 s at 6000 rpm on a Precellys 24-Dual homogenizer (Bertin Technologies, Montigny le Bretonneux, France). Further DNA extraction was performed with the QIAamp DNA Stool mini kit (Qiagen) in accordance with the manufacturer’s instructions. DNA integrity was evaluated by loading 3 μL of DNA on a 0.8% agarose gel stained with ethidium bromide. The purity and concentration of the extracted DNA were measured using ultraviolet absorption at 260/280 nm and 230/280 nm ratio (NanoDrop 1000 spectrophotometer, Thermo Scientific, Waltham, MA, USA).

Denaturing gradient gel electrophoresis (DGGE) separates DNA fragments of the same length but with different base-pair sequences. DNA fragments were generated from the small intestinal content DNA samples applying community PCR with universal bacterial primers targeting the variable V3 region of the 16S ribosomal RNA. The nucleotide sequences of the primers were as follows: forward primer F341 with GC clamp 5’-CGC CCG CCG CGC GCG GCG GGC GG GCG GGG GCA CGG GGGG - CCT ACG GGA GGC AGC AG 3’ and reverse primer R518 5’ATT ACC GCG GCT GCT GG-3’ [35]. PCR amplification was performed in duplicate using a Mastercycler Gradient (Eppendorf, Hamburg, Germany), and each PCR reaction was done in a 45 μL total reaction mixture using 3 μL of the DNA sample (4 ng/μL), 0.125 μM of each of the primers, 100 μM deoxynucleotide triphosphate (dNTP) (Peqlab Biotechnologie GmbH, Erlangen, Germany), and 0.6 μL of peqGOLD Taq-DNA-Polymerase (5 U/μL) (Peqlab). The PCR conditions used were 1 cycle of 94 °C for 5 min, followed by 9 cycles of 94 °C for 30 s, 64 °C for 40 s (decreased by 0.5 °C/cycle) and 72 °C for 40 s. Subsequently 19 cycles of 94 °C for 30 s, 56 °C for 40 s and 72 °C for 40 s, followed by one cycle of 72 °C for 4 min, were run. DGGE was performed as described by [35] with the INGENYPhorU-2×2 system (Ingeny, Goes, The Netherlands). Briefly, amplicons were separated using a 30 to 60% denaturating gradient [35]. 30 μL of the PCR product was loaded and electrophoresis was performed at 100 V for 16 h at 60 °C. Each gel included four standard reference lanes containing amplicons of 12 bacterial species for normalization and comparison between gels. DGGE gels were stained with 1x SYBR Green I (Sigma-Aldrich) for 30 min. Fingerprinting profiles were visualized using the Bio Vision Imaging system (Peqlab) and the Vision-Capt software (Vilber Lourmat, Marne-la-Vallée, France). The microbial profiles were processed with GelCompar II vs. 6.6 (Applied Maths NV, Sint-Martens-Latem, Belgium).

The similarity between DGGE-profiles, given as a percentage, was analyzed using the Dice similarity coefficient, derived from presence or absence of bands. On the basis of a distance matrix, which was generated from the similarity values, dendrograms were constructed using the unweighted pair group method with arithmetic means (UPGMA) as clustering-method. The microbial richness (R) was assessed as the number of bands within a profile.

Low-GC-containing operational taxonomic units (OTUs) were selected for identification based on the differences between the DGGE patterns of the control group compared to the FBs contaminated group. After extraction of the selected bands, and reapplication on DGGE to confirm their positions relative to the original sample, the respective 16S-fragments were sequenced (LGC Genomics, Berlin, Germany) and aligned to the NCBI GenBank prokaryotic 16S ribosomal RNA database using the standard nucleotide BLASTN 2.2.30+ (nucleotide basic local alignment search tool) [36].

### Evaluation of the consequences of FBs exposure on necrotic enteritis

#### Quantification of total C. perfringens by qPCR

Total *C. perfringens* in ileal content samples, collected before experimental *C. perfringens* challenge (day 15), was quantified using the *cpa* gene (encoding alpha toxin) as target gene. qPCR was performed using SYBR-green 2x master mix (Bioline, Brussels, Belgium) in a Bio-Rad CFX-384 system. Each reaction was done in triplicate in a 12 μL total reaction mixture using 2 μL of the DNA sample and 0.5 μM final qPCR primer concentration (Table 2). The qPCR conditions were 1 cycle of 95 °C for 10 min, followed by 40 cycles of 95 °C for 30 s, 60 °C for 30 s, and stepwise increase in the temperature from 65° to 95 °C (at 10s/0.5 °C). Melting curve data were analyzed to confirm the specificity of the reaction. For construction of the standard curve, the PCR product was generated using the standard PCR primers (Table 2) and DNA from *C. perfringens* strain CP56. After purification (MSB Spin PCRapace, Stratec Molecular, Berlin, Germany) and determination of the DNA concentration with a Nanodrop ND 1000 spectrophotometer (Nanodrop Technologies, Wilmingtom, DE, USA), the concentration of the linear dsDNA standard was adjusted to 1 × 10^8^ to 1 × 10^1^ copies per μL with each step differing by 10 fold. The copy numbers of samples (copies/g intestinal content) were determined by reading off the standard series with the Ct values of the samples.

**Table 2:** **Primer sequences used for (q)PCR analyses**

Target	Forward primer	Reverse primer	Analysis	Reference
*cpa*	AGT CTA CGC TTG GGA TGG AA	TTT CCT GGG TTG TTC ATT TC	PCR	[55]
*cpa*	GTT GAT AGC GCA GGA CAT GTT AAG	CAT GTA GTC ATC TGT TCC AGC ATC	qPCR	[56]
*netB*	TGA TAC CGC TTC ACA TAA AGG T	ACC GTC CTT AGT CTC AAC AAA T	PCR	[30]
*netB*	TCA ATT GGT TAT TCT ATA GGC GGT A	ATA TGA AGC ATT TAT TCC AGC ACC A	qPCR	[57]
*rpoA*	ACA TCA TTA GCG TTG TCA GTT AAA G	GAG GTT ATG GAA TAA CTC TTG GTA ATG	PCR	[30]
*rpoA*	CCA TCT GTT TTT ATA TCT GCT CCA GTA	GGA AGG TGA AGG ACC AAA AAC TAT T	qPCR	[57]

Sequences are presented from 5’ to 3’.

#### C. perfringens infection trial

The remaining 28 animals per pen were used in a *C. perfringens* experimental infection trial as previously described [30]. The BW of all animals was measured on day 16 and day 21. Gumboro vaccine (Nobilis Gumboro D78, MSD Animal Health, Brussels, Belgium) was administered on day 16 in the drinking water of all cages. Both groups were experimentally infected with an oral bolus of 4.10^8^ cfu *C. perfringens* strain 56 on days 17, 18, 19 and 20. On day 21, 22 and 23, each day one third of each group was euthanized by overdose sodium pentobarbital and immediately submitted to necropsy. Macroscopic NE lesion scoring of the small intestines (duodenum, jejunum and ileum) was performed single-blinded as follows; 0 no gross lesions; 1 small focal necrosis or ulceration (one to five foci); 2 focal necrosis or ulceration (six to 15 foci); 3 focal necrosis or ulceration (16 foci or more); 4 patches of necrosis of 2 to 3 cm long; 5 diffuse necrosis typical field cases, partially adapted from [37]. Chickens with a lesion score of 1 or more were classified as NE positive.

#### In vitro assessment of the effect of FB_1_ on C. perfringens growth, and cpa and netB transcription

Following concentrations of FB_1_ were tested for their effect on *C. perfringens* growth and toxin production: 0, 0.2, 2 and 20 μg FB_1_/mL. All tests were performed in triplicate.

The *C. perfringens* inoculum was 1:1000 diluted in TGY medium, containing the different concentrations of FB_1_, and incubated anaerobically at 37 °C. A growth curve was produced by bacterial plating of a ten-fold dilution series of the culture at 0, 2, 3, 4, 5, 6, 7, 8 and 24 h after inoculation. Ten-fold dilutions were prepared in phosphate buffered saline (PBS), and subsequently plated on Colombia agar with 5% sheep blood. After anaerobic incubation overnight at 37 °C, the number of colony forming units (cfu)/mL was determined.

The impact of FB_1_ on *cpa* (alpha toxin) and *netB* (netB toxin) transcription was tested by qRT-PCR. The *C. perfringens* inoculum was 1:10 000 diluted in TGY medium, containing the different concentrations of FB_1_, and incubated anaerobically at 37 °C until an optical density (OD) of 0.6-1.0 was measured at a wavelength of 600 nm (6h of incubation). The transcription levels of *cpa* and *netB* in the presence of FB_1_ were compared to non-FB_1_ contaminated test conditions and normalized to the housekeeping gene *rpoA*, encoding RNA polymerase subunit A. Total RNA was isolated using SV total RNA Isolation system (Promega, Leiden, The Netherlands). RNA was treated with Turbo DNA-free kit (Ambion, Austin, TX, USA) per the manufacturer’s instructions to remove genomic DNA contamination. Subsequently, RNA was converted to cDNA with iScript cDNA Synthesis Kit (Bio-Rad, Temse, Belgium). qRT-PCR was performed using SYBR-green 2x master mix (Bioline, Brussels, Belgium) in a Bio-Rad CFX-384 system. Each reaction was done in triplicate in a 12 μL total reaction mixture using 2 μL of cDNA sample and 0.5 μM final qPCR primer concentration (Table 2). The qPCR conditions were as described above for total *C. perfringens* determination in ileal content samples. For construction of the standard curve, the PCR product was generated using the standard PCR primers (Table 2).

### Statistical analyses

Statistical program SPSS version 22 was used for data analysis. To compare the number of NE positive birds (lesion score ≥2) between different groups, binomial logistic regression was used. All other parameters, including BW relative organ weight, length of small intestines, Sa/So ratio, villus height/crypt depth measurements, concentration of *C. perfringens* in ileal digesta, in vitro assessment of clostridial growth, and *cpa* and *netB* transcription were analyzed by an independent Student’s t-test, after determination of normality. Significance level was set at 0.05.

## Results

### FBs negatively affect sphingolipid metabolism

The inhibition of ceramide synthase by FBs causes an intracellular accumulation of sphingoid bases, mainly sphinganine. An increased Sa/So is suggested to be the most sensitive biomarker to FBs intoxication in many animal species [2]. The plasma Sa/So ratio was 1.5 fold higher in animals fed the FBs contaminated diet compared to the control animals, 0.21 ± 0.016 versus 0.14 ± 0.014, respectively (*P <* 0.001). No significant differences were observed in BW between the control group and the FBs contaminated group (Table 3). A trend (*P =* 0.060) was observed for an increased relative weight of liver in chickens fed the FBs contaminated diet (3.69 ± 0.134% of BW) compared to the control group (3.39 ± 0.081% of BW). Relative weight of bursa, spleen, proventriculus, ventriculus, kidneys, lungs and heart did not differ between both experimental groups (data not shown).

**Table 3:** **Bodyweight of broiler chickens measured on day 1, 8, 16 and 21**

		Day 1	Day 8	Day 16	Day 21
		BW (g)			
Control diet	♂	43 ± 3 (*n* = 52)	161 ± 22 (*n* = 52)	488 ± 78 (*n* = 43)	794 ± 134 (*n* = 43)
	♀	44 ± 4 (*n* = 50)	153 ± 21 (*n* = 50)	448 ± 53 (*n* = 41)	754 ± 88 (*n* = 41)
FBs contaminated diet	♂	43 ± 3 (*n* = 51)	176 ± 26 (*n* = 51)	519 ± 65 (*n* = 42)	841 ± 88 (*n* = 42)
	♀	43 ± 3 (*n* = 51)	168 ± 23 (*n* = 51)	476 ± 58 (*n* = 42)	785 ± 104 (*n* = 42)

Animals were fed a control diet or a FBs contaminated diet (18.6 mg FB_1_+FB_2_/kg feed). Data presented as mean bodyweight (BW) (g) ± SEM. *n* = number of animals.

### FBs reduce total small intestinal length, ileal villus height and crypth depth

The total length of the small intestine was significantly (*P =* 0.033) decreased in birds of the FBs contaminated group compared to the control group (130.5 ± 2.37 and 139.0 ± 2.95 cm, respectively). No differences were observed in the relative percentage of length of the different segments of the small intestine (Table 4).

**Table 4:** **Length of small intestinal segments**

Organ	Control diet	FBs contaminated diet	P
Total length small intestines^a^ (cm)	139.0 ± 2.95	130.5 ± 2.37	0.033*
Duodenum (% of total length)^b^	16.9 ± 0.40	17.2 ± 0.35	0.656
Jejunum (% of total length)^b^	43.1 ± 0.69	41.7 ± 0.45	0.118
Ileum (% of total length)^b^	40.0 ± 0.71	41.1 ± 0.37	0.184

Animals were randomly divided in two experimental groups, each group consisting of three pens. One group was fed a control diet and one was fed a FBs contaminated diet (18.6 mg FB_1_+FB_2_/kg feed). Six birds (3♂/3♀) per pen were euthanized on day 15 and the length of small intestinal segments was recorded.

Data presented as mean ± SEM.

^a^Total length small intestines including all three segments: duodenum, jejunum and ileum; ^b^% of total length = (length segment (cm)/total length small intestines (cm)) × 100; (*) significantly different (*P* < 0.05)/trend (*P* < 0.10).

Feeding a FBs contaminated diet significantly reduced villus height (*P =* 0.002) and crypt depth (*P =* 0.011) in ileum (Table 5). No effect was observed on ileal villus to crypt ratio. No effect was shown in duodenum and jejunum.

**Table 5:** **Effect of FBs on villus height and crypt depth**

	Control diet	FBs contaminated diet	P
Mid-duodenum			
Villus height (μm)	1628 ± 39.4	1549 ± 44.8	0.225
Crypt depth (μm)	196 ± 7.1	197 ± 7.8	0.999
Villus to crypt ratio	8 ± 0.3	8 ± 0.4	0.337
Mid-jejunum			
Villus height (μm)	842 ± 32.0	880 ± 28.6	0.278
Crypt depth (μm)	173 ± 6.4	182 ± 6.5	0.385
Villus to crypt ratio	5 ± 0.2	5 ± 0.2	0.927
Mid-ileum			
Villus height (μm)	497 ± 31.7	393 ± 16.2	0.002*
Crypt depth (μm)	155 ± 4.4	131 ± 5.2	0.011*
Villus to crypt ratio	3 ± 0.2	3 ± 0.1	0.349

Broiler chickens were fed a control diet or a FBs contaminated diet (18.6 mg FB_1_+FB_2_/kg feed) for 15 days. Samples of different small intestinal segments of six birds (3♂/3♀) per pen, three pens per group, were collected at day 15. Analysis based on mean of 10 measurements per segment per animal was calculated; data are presented as weighted mean ± SEM. (*) significantly different (*P* < 0.05).

### FBs affect the ileal microbiota composition

**Figure 1:**
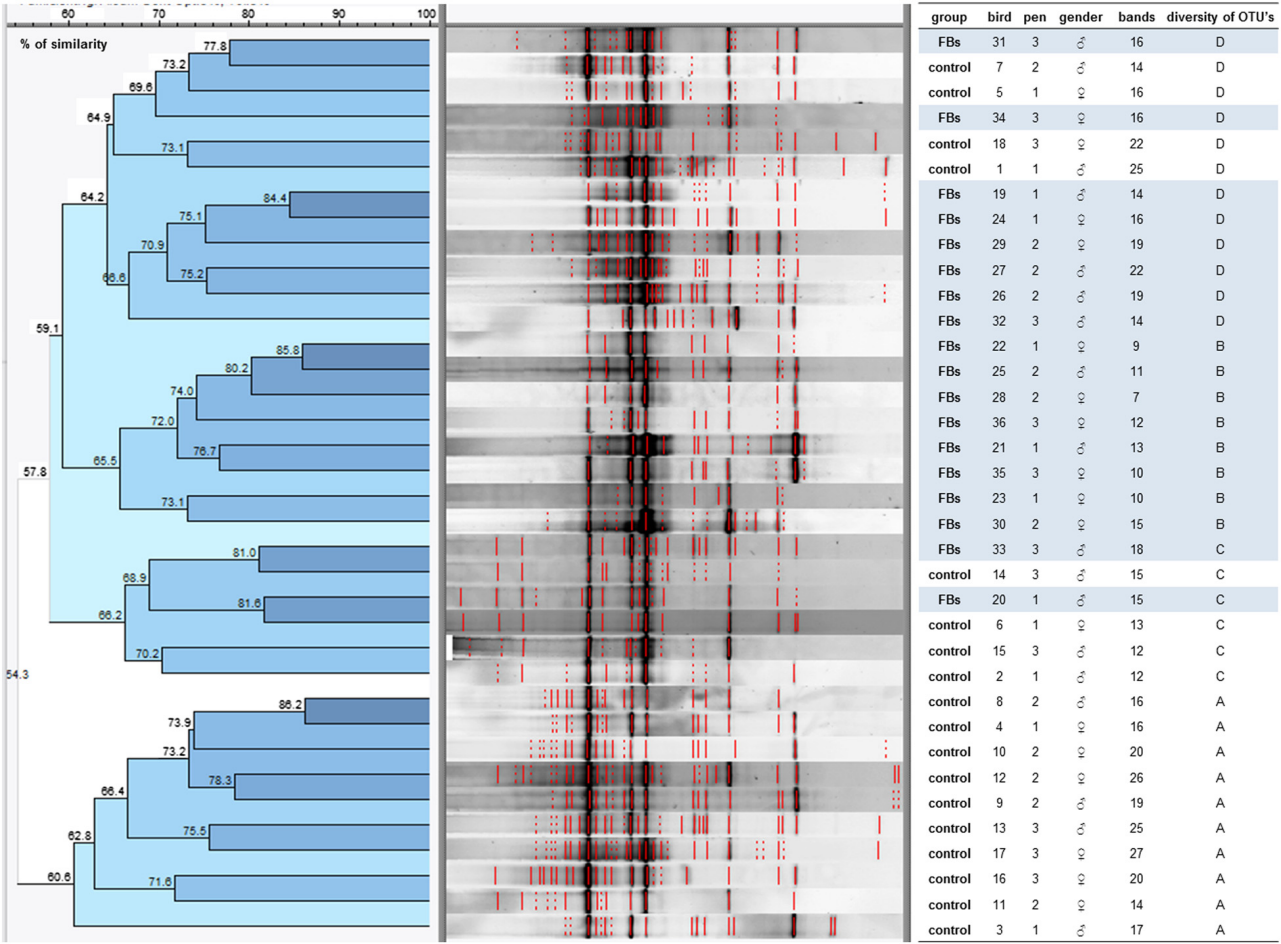
**Denaturing gradient gel electrophoresis (DGGE) fingerprint of DNA samples of ileal content applying community PCR with universal bacterial primers targeting the variable V3 region of the 16S ribosomal RNA (18 animals per group (3 pens/group, 6 animals/pen)).** Percentage of similarity between DGGE profiles was analyzed using the Dice similarity coefficient, derived from presence or absence of bands. On the basis of a distance matrix, which was generated from the similarity values, dendrograms were constructed using the unweighted pair group method with arithmetic means (UPGMA) as clustering-method. The microbial richness (R) was assessed as the number of OTUs within a profile. Within ileum-samples a separation according to the treatment is visible, ^(D)^although exceptions occur like samples of chicken 20, 31, 33 and 34 of FBs group which show high similarity (69.6 – 81%) with control group. ^(A)^The majority of the control-samples contains bands in the lower GC-range which ascribes them to one clade. ^(B)^Among the FBs group a clade of clearly reduced diversity is formed by 8 samples. ^(C)^The remaining of this treatment group shows a similar diversity of OTUs of medium GC-content like the control-group, however the low-GC-OTUs were absent.

DGGE fingerprint of DNA samples of duodenal and jejunal content, applying community PCR with universal bacterial primers targeting the variable V3 region of the 16S ribosomal DNA, could not show a difference in microbiota composition between chickens fed the control diet or the FBs contaminated diet. In the duodenum, three clades were observed related to the diversity of OTUs, independent of the treatment. One clade showed five samples with a reduced number of bands in the duodenum. Most samples had an average diversity between 10 and 15 OTUs across the medium GC-range. Some samples consisted of 18 to 31 OTUs of the medium and high GC-range (Additional file 1). No difference in number of OTUs between both experimental groups was shown in the jejunum. OTUs were all located in the medium range of GC-content (Additional file 2). Within ileum content samples a difference of the DGGE fingerprint according to the treatment was seen. The majority of the ileum samples of the control group contained OTUs in the lower GC-range which ascribes them to one clade (Figure 1, clade A). Among the FBs group a clade of clearly reduced diversity is formed by eight samples (Figure 1, clade B). Another group of samples of this treatment group show a similar diversity of OTUs of medium GC-content compared to the control-group, but the low-GC-OTUs were absent (Figure 1, clade C). The dendrogram of DGGE profiles of four chickens (bird 20, 31, 33 and 34) of the FBs group showed a high similarity with the control group (Figure 1, clade D).

Subsequently, five low-GC-content OTUs from ileal content samples of the control group, which made the difference in their DGGE-patterns compared to the FBs group, were identified at genus level by sequencing. Based on these results the most affected groups were related to the genera *Clostridium* and *Lactobacillus*. OTUs 19 and 20 had 99.09-100% sequence similarity with the type strain of *Candidatus* Arthromitus, recently renamed as *Candidatus* Savagella [38]. OTUs 4 and 13 had 100% sequence similarity with *Lactobacillus johnsonii* and, OTU 16 was similar to the sequence of an unknown species of the genus *Lactobacillus*.

### FBs increase the susceptibility for *C. perfringens* induced necrotic enteritis

Quantification of total *C. perfringens* in DNA samples of ileal content (day 15 of animal trial) by qPCR using *cpa* gene (alpha toxin) showed an increased level in chickens fed a FBs contaminated diet compared to a control diet (7.5 ± 0.30 versus 6.3 ± 0.24 log10 copies/g intestinal content) (*P* = 0.027).

**Figure 2:**
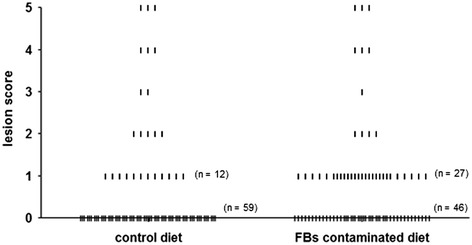
**NE lesion score of individual broiler chickens challenged with** ***C. perfringens.*** Chickens were fed either a control diet or a FBs contaminated diet. Subsequently, birds were orally inoculated with *C. perfringens* strain 56. Macroscopic intestinal NE lesions in the small intestine (duodenum to ileum) were scored as follow; 0 no gross lesions; 1 small focal necrosis or ulceration (one to five foci); 2 focal necrosis or ulceration (six to 15 foci); 3 focal necrosis or ulceration (16 or more); 4 patches of necrosis of 2 to 3 cm long; 5 diffuse necrosis typical field cases. Chickens with NE lesions scores of 1 or more were categorized as NE positive. No effect was observed on the mean lesion scores of NE positive chickens.

The number of chickens with NE increased from 29.8 ± 5.46% of the birds in the control group to 44.9 ± 2.22% of broilers which were fed the FBs contaminated diet (*P* = 0.047). No effect was observed on the mean lesion scores of NE positive broiler chickens (Figure 2). No macroscopic coccidiosis lesions were observed.

Results of the *C. perfringens* growth assay showed no influence of different concentrations of FB_1_ on the bacterial growth curve in vitro (data not shown). qRT-PCR analyses showed no impact of FB_1_ on transcription of genes encoding alpha (*cpa*) and netB (*netB*) toxin. Results for the *cpa* and *netB* gene transcription were normalized to the *rpoA* gene. The relative *cpa* transcription (log copies *cpa*/log copies *rpoA*) was 1.21 ± 0.008, 1.21 ± 0.006, 1.21 ± 0.004, and 1.22 ± 0.011 in the presence of 0, 0.2, 2 or 20 μg FB_1_/mL, respectively. The relative *netB* transcription (log copies *netB*/log copies *rpoA*) was 1.03 ± 0.016, 1.05 ± 0.013, 1.04 ± 0.015, and 1.04 ± 0.007 in the presence of 0, 0.2, 2 or 20 μg FB_1_/mL, respectively.

## Discussion

The ingestion of FBs contaminated feed by broiler chickens, at a level of about 20 mg FB_1_+FB_2_/kg feed, affects the intestinal microbial homeostasis. Subsequently, these changes possibly predispose the birds to *C. perfringens* induced NE. To our knowledge, this is the first time such an effect has been demonstrated.

FBs negatively affect broiler health, demonstrated by the increased plasma Sa/So ratios in broiler chicks fed a FBs contaminated diet. These results suggest that the sphingolipid metabolism was impaired after exposure to levels of FBs approaching the EU maximum guidance levels [31]. FBs inhibit the ceramide synthase enzyme, causing an intracellular accumulation of sphingoid bases, mainly sphinganine. Since the disruption in the sphingolipid metabolism occurs before other indicators of cell injury, the Sa/So ratio is suggested to be the most sensitive biomarker to FB intoxication in many animal species [13,39]. A similar increase in the Sa/So ratio has been demonstrated in serum of broilers fed 80–100 mg FB_1_/kg feed for 3–4 weeks [14,40,41]. Furthermore, a linear dose-dependent increase in the Sa/So ratio has been observed in the liver of broiler chickens fed 20–80 mg FB_1_/kg feed for three weeks [40]. In the present study, the relative weight of liver was increased in broilers fed the FBs contaminated diet. A similar effect has already been observed when broiler chickens were fed a FBs contaminated diet containing 100 mg FB_1_ and 20 mg FB_2_/kg feed for 2–4 weeks [14]. In broilers, this effect was not reported in other studies using low levels FB_1_ (<100 mg/kg feed) [8,40]. Dietary exposure to FBs has been associated with histopathological degenerative changes in the hepatocytes including mild vacuolar degeneration and bile duct hyperplasia [42].

Consumption of a diet contaminated with FBs for 15 days reduced small intestinal length, ileal villus height and crypt depth. These results could be related to the negative impact of FB_1_ on epithelial cell proliferation, reducing villus renewal and impairing intestinal absorption of nutrients [13]. This is in accordance with a previous study, where a decreased villus height was observed in the jejunum of broiler chickens fed high concentrations of FBs (>100 mg FB_1_/kg feed) [14,15]. In pigs exposed to FBs for 9 days (1.5 mg FB_1_/kg BW), however, ileal villi tended to be longer [43]. It remains to be determined if this effect on intestinal morphology is induced only by a direct toxic effect of FBs on intestinal epithelial cells, or also indirectly, by the microbiota shift induced by FBs. Longer villi are for example observed in the ileum of chickens treated with *L. reuteri,* indicating that the composition of the intestinal microbiota may indeed affect intestinal morphology [44]. Since the intestinal mucus layer and microbiota are strongly associated, FBs could also modify the microbiota through modulation of the mucus production. Goblet cell hyperplasia was observed in broiler chickens exposed to very high dietary concentration of FB_1_ (300 mg/kg feed) for two weeks [15]. Similarly, it was demonstrated that non-cytotoxic concentrations of DON decreased mucin production in human colonic epithelial goblet cells (HT-29 16E) and porcine intestinal explants [45]. The ingestion of FBs contaminated feed by broiler chickens for 15 days resulted in a modified composition of the intestinal microbiota of the ileum. Based on separation of DNA fragments by electrophoresis of PCR-amplified 16S ribosomal DNA fragments, using polyacrylamide gels containing a linear gradient of DNA denaturants, the DGGE technique provides a genetic fingerprint of a complex microbial community. The PCR product banding pattern is indicative of the number of bacterial species or assemblages of species that are present [35]. The results clearly indicate a reduced diversity of the ileal microbiota in broiler chickens exposed to FBs compared to the control group. The difference was mainly due to a reduced presence of low-GC-content OTUs in ileal content samples of FBs exposed animals. Feeding a FBs contaminated diet to broiler chickens was correlated with a decrease in the abundance of *Candidatus* Arthromitus, recently renamed to *Candidatus* Savagella [38]. These segmented filamentous bacteria (SFB) are a unique group of uncultivated commensal bacteria within the bacterial family of *Clostridiaceae*. These SFB are characterized by their attachment to the intestinal epithelium and their important role in modulating host immune systems [38,46]. They induce IgA secreting cells and influence the development of the T-cell repertoire [47,48]. Stanley et al. [47] demonstrated that the best-known predisposing factor for necrotic enteritis, coccidiosis, also eliminates or reduces the levels of this immune modulating bacterium. Since coccidiosis and FBs are both predisposing factors for *C. perfringens* induced NE in broiler chickens, the role of *Candidatus* Savagella in the pathogenesis of NE needs to be further investigated. It has been suggested that the colonization of the ileum with SFB is correlated with the population of lactobacilli [49]. Lactobacilli belong to the low GC Gram-positive group of Lactobacillales, fermenting sugars to lactic acid [50]. In this study, FBs also modulated the presence of *Lactobacillaceae* in the ileum. *L. johnsonii* (OTUs 4 and 13 with sequence similarity of 100%) was reduced in FBs exposed birds. *L. johnsonii* has been extensively investigated for its probiotic activities including pathogen inhibition, epithelial cell-attachment, and immunomodulation [50]. Similar to our results, it was recently demonstrated that *L. johnsonii* was reduced in birds fed fishmeal with or without *C. perfringens* challenge [47,51]. A positive association was demonstrated between crude protein derived from fishmeal and numbers of ileal and caecal *C. perfringens* [52]. *L. johnsonii* interferes with the colonization and persistence of *C. perfringens* in poultry [53] and some lactobacilli can inhibit growth of *C. perfringens* [54]. It needs to be further investigated whether lactic acid bacteria (LAB) are able to counteract the negative effects of mycotoxins on the intestinal health in poultry.

In conclusion, feeding a FBs contaminated diet at contamination levels approaching the EU maximum guidance level altered the sphingolipid metabolism in broiler chickens without affecting BW gain. FBs modified the composition of the intestinal microbiota of the ileum. DGGE analysis demonstrated a reduced presence of low-GC content OTUs in ileal digesta of birds exposed to FBs, which were subsequently identified as a reduced abundance of *Candidatus* Savagella and *Lactobaccilus* spp. such as *L. johnsonii*. The ileal concentration of total *C. perfringens* was increased in chickens fed the FBs contaminated diet. Additionally, small intestinal length, ileal villus height, and crypt depth were negatively affected by FBs. The changes in the gut microbiota possibly induced an environment stimulating *C. perfringens* colonization, and predisposing the birds to necrotic enteritis. The impact of different predisposing factors for NE in broilers, among others coccidiosis, fishmeal and FBs, on intestinal microbiota shows remarkable similarities. The observed predisposing effect is due to the negative impact of FBs on the intestinal microbiota and the animal host, rather than its effect on the bacterium itself.

## Additional files

Additional file 1:
**Denaturing gradient gel electrophoresis (DGGE) fingerprint of DNA samples of duodenal content applying community PCR with universal bacterial primers targeting the variable V3 region of the 16S ribosomal RNA (18 animals per group (3 pens/group, 6 animals/pen)).** Percentage of similarity between DGGE profiles was analyzed using the Dice similarity coefficient, derived from presence or absence of bands. On the basis of a distance matrix, which was generated from the similarity values, dendrograms were constructed using the unweighted pair group method with arithmetic means (UPGMA) as clustering-method. The microbial richness (R) was assessed as the number of OTUs within a profile. Treatment is not reflected by DGGE fingerprint. Independently of treatment three clades are distinguishable concerning the diversity of OTUs: ^(A)^one clade with reduced number of bands built by 2 FBs-samples and 3 control-samples, a second clade ^(B)^with average diversity between 10 and 15 OTUs across the medium GC-range and a third clade ^(C)^consisting of 18 to 31 OTUs again in the medium but also high GC-range. Additional file 2:
**Denaturing gradient gel electrophoresis (DGGE) fingerprint of DNA samples of jejunal content applying community PCR with universal bacterial primers targeting the variable V3 region of the 16S ribosomal RNA (18 animals per group (3 pens/group, 6 animals/pen)).** Percentage of similarity between DGGE profiles was analyzed using the Dice similarity coefficient, derived from presence or absence of bands. On the basis of a distance matrix, which was generated from the similarity values, dendrograms were constructed using the unweighted pair group method with arithmetic means (UPGMA) as clustering-method. The microbial richness (R) was assessed as the number of OTUs within a profile. Treatment is not reflected by DGGE fingerprint. ^(A)^No difference in number of OTUs was demonstrated. In general, all samples OTUs were located in the medium range of GC-content. ^(1)^The banding-pattern of bird 8, control group, is shifted to the lower GC-range what flags it unique in comparison to the others which hardly exhibit bands in this area.

## Abbreviations

BW: Bodyweight
DGGE: Denaturing gradient gel electrophoresis
dNTP: Deoxynucleotide triphosphate
DON: Deoxynivalenol
ELEM: Equin leucoencephalomalacie
FB_1_: Fumonisin B_1_
FBs: Fumonisins
IFN: Interferon
IL: Interleukin
LC-MS/MS: Liquid chromatography-tandem mass spectrometry method
NE: Necrotic enteritis
OD: Optical density
OTUs: Operational taxonomic units
PBS: Phosphate buffered saline
PPE: Porcine pulmonary edema
R: Microbial richness
Sa: Sphinganine
So: Sphingosine
TGY: Tryptone glucose yeast
V:C: Villus-to-crypt ratio
VH: Villus height
