# Supplementary figures and images for: Fumonisins affect the intestinal microbial homeostasis in broiler chickens, predisposing to necrotic enteritis
Source: Vet Res. 2015 Sep 23;46(1):98. doi: 10.1186/s13567-015-0234-8 (PMC4579638; doi:10.1186/s13567-015-0234-8)

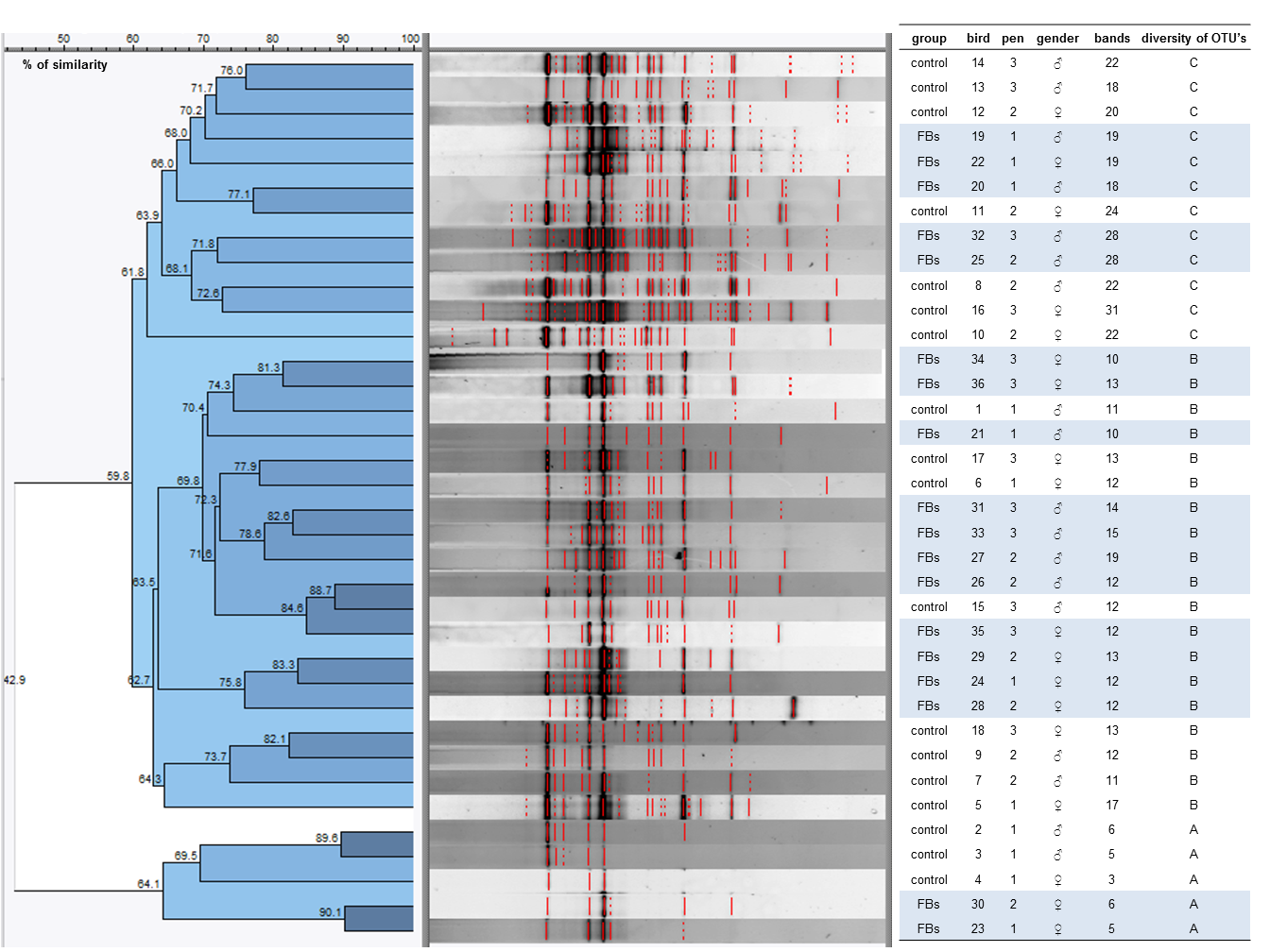

Supplement: Additional file 1: — Denaturing gradient gel electrophoresis (DGGE) fingerprint of DNA samples of duodenal content applying community PCR with universal bacterial primers targeting the variable V3 region of the 16S ribosomal RNA (18 animals per group (3 pens/group, 6 animals/pen)). Percentage of similarity between DGGE profiles was analyzed using the Dice similarity coefficient, derived from presence or absence of bands. On the basis of a distance matrix, which was generated from the similarity values, dendrograms were constructed using the unweighted pair group method with arithmetic means (UPGMA) as clustering-method. The microbial richness (R) was assessed as the number of OTUs within a profile. Treatment is not reflected by DGGE fingerprint. Independently of treatment three clades are distinguishable concerning the diversity of OTUs: (A)one clade with reduced number of bands built by 2 FBs-samples and 3 control-samples, a second clade (B)with average diversity between 10 and 15 OTUs across the medium GC-range and a third clade (C)consisting of 18 to 31 OTUs again in the medium but also high GC-range. [file 13567_2015_234_MOESM1_ESM.tif]

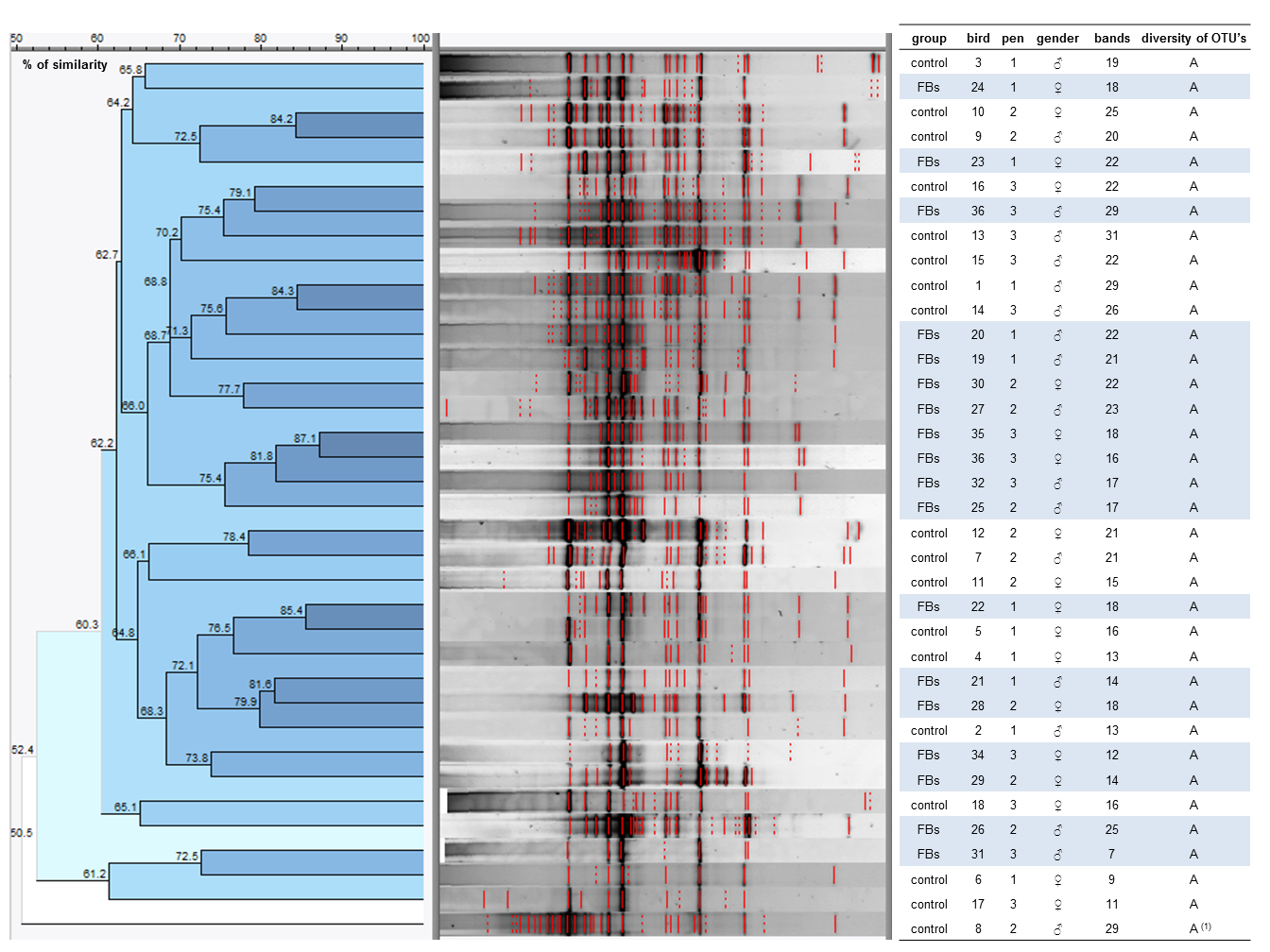

Supplement: Additional file 2: — Denaturing gradient gel electrophoresis (DGGE) fingerprint of DNA samples of jejunal content applying community PCR with universal bacterial primers targeting the variable V3 region of the 16S ribosomal RNA (18 animals per group (3 pens/group, 6 animals/pen)). Percentage of similarity between DGGE profiles was analyzed using the Dice similarity coefficient, derived from presence or absence of bands. On the basis of a distance matrix, which was generated from the similarity values, dendrograms were constructed using the unweighted pair group method with arithmetic means (UPGMA) as clustering-method. The microbial richness (R) was assessed as the number of OTUs within a profile. Treatment is not reflected by DGGE fingerprint. (A)No difference in number of OTUs was demonstrated. In general, all samples OTUs were located in the medium range of GC-content. (1)The banding-pattern of bird 8, control group, is shifted to the lower GC-range what flags it unique in comparison to the others which hardly exhibit bands in this area. [file 13567_2015_234_MOESM2_ESM.tif]
